# Uncovering the Secret of Mesenchymal Stromal Cells Secretome: From Extracellular Vesicle Cargo to Neuroprotection

**DOI:** 10.3390/cells15100889

**Published:** 2026-05-13

**Authors:** Michael Joseph, Martina Gabrielli, Elisa Tonoli, Gareth W. V. Cave, Elisabetta A. M. Verderio

**Affiliations:** 1 School of Science and Technology, Nottingham Trent University, Nottingham NG11 8NS, UK; michael.2023@my.ntu.ac.uk (M.J.); martina.gabrielli02@ntu.ac.uk (M.G.); elisa.tonoli@ntu.ac.uk (E.T.); gareth.cave@ntu.ac.uk (G.W.V.C.); 2 BIGEA Biological Sciences, University of Bologna, 40126 Bologna, Italy

**Keywords:** mesenchymal stromal cells (MSCs), extracellular vesicles (EVs), cargo, proteome, miRNAs, lipids, neurodegeneration, blood–brain barrier (BBB), clinical trials, neuroprotection, brain

## Abstract

**Highlights:**

**What are the main findings?**

- The molecular cargo of mesenchymal stromal cell-derived extracellular vesicles (MSC-EVs) reported in the recent literature was mapped by integrating proteomic, transcriptomic, and lipidomic evidence across different MSC sources.
- The neuroprotective potential of MSC-EVs, routes of delivery to the brain, and progress in MSC-EV-based clinical trials are comprehensively reviewed.

**What are the implications of the main findings?**

- The conserved MSC-EV cargo is enriched in anti-inflammatory and immunomodulatory proteins, regulatory miRNAs, and extracellular matrix components associated with cell survival and tissue repair, compatible with neuroprotective functions.
- Comprehensive mapping of MSC-EV cargo provides mechanistic insights into their therapeutic potential in a rapidly evolving translational field.

**Abstract:**

Mesenchymal stromal cells (MSCs), also known as multipotent stromal cells or mesenchymal stromal cells, support cell growth and viability through the secretion of trophic factors and immunomodulatory molecules. Their secretome exerts cytoprotective effects in the brain, although the mechanisms underlying MSC-mediated neurological recovery remain poorly understood. A substantial portion of the MSC secretome is delivered via extracellular vesicles (EVs), membrane-bound particles that facilitate intercellular communication. EVs derived from MSCs of various origins exhibit therapeutic potential, and numerous studies are examining the miRNA and protein cargo contained within MSC-EVs. Despite these efforts, methodological differences across the literature and the inherent variability associated with MSC sources have limited data interpretation and identification of EV-factors which may be responsible for neuroprotection. In this study, we have reviewed proteomic, transcriptomic and lipidomic datasets from a selection of recent MSC-EV studies, to identify shared cargo components that may contribute to promoting cell repair and plasticity in brain, counteracting neurodegeneration.

## 1. Mesenchymal Stromal Cells: Historical Perspective

The concept of mesenchymal stromal/stem cells (MSCs) originates from pioneering studies by Friedenstein and co-workers in the 1960s and 1970s, who were the first to report that adult bone marrow contains not only hematopoietic cells but also a distinct population of fibroblast-like adherent cells in the stromal compartment with the potential to differentiate into bone and connective tissues [1]. It was established that these osteogenic cells are unique and could regenerate [1], laying the foundation for the identification and characterisation of the osteogenic stem cells within bone marrow, work that led to the introduction of the term “bone marrow stromal cells”. In the 90s, Caplan expanded the concept proposing the ‘mesengenic process’, the capacity of these cells to give rise to multiple connective tissue phenotypes, including bone, cartilage, and adipose tissue [2]. Initially, it was believed that these MSCs could replace injured cells and regenerate new tissues by direct differentiation [2,3]; however, subsequent evidence revealed that the mechanism of action is mediated through paracrine and immunomodulatory effects, driven by the secretion of bioactive factors such as, cytokines, and growth factors and extracellular vesicles collectively known as the secretome [4].

## 2. Characteristics of MSCs

MSCs are defined by a set of minimal criteria originally proposed by the International Society for Cell & Gene Therapy (ISCT). These include plastic adherence under standard culture conditions and a fibroblast-like morphology, and a characteristic immunophenotype. Specifically, MSCs express the surface markers CD73, CD90, and CD105, while showing lack of haematopoietic markers such as CD34, CD45, CD14 or CD11b, CD79α or CD19, and HLA-DR [5]. In addition to their phenotypic profile, MSCs are characterised by their multilineage differentiation capacity, which encompasses osteogenic, adipogenic, and chondrogenic lineages in vitro—features that underpin their proposed roles in tissue repair and regeneration [6]. Differentiation is regulated by a complex interplay of extrinsic and intrinsic factors including growth factors, transcription factors, cytokines [7,8] and epigenetic modifications like DNA methylation and histone alterations which collectively modulate gene expression programmes governing MSC fate decisions (as reviewed by Perez-Campo et al. [9]). In accordance with current consensus, the term *MSC* is used here to denote mesenchymal stromal cells and does not imply stemness (self-renewal and in vivo multipotency), unless self-renewal and in vivo differentiation potential are explicitly demonstrated.

However, despite established minimal criteria, the definition and characterisation of MSCs remain in evolution. Significant heterogeneity in proteomic and transcriptomic profiles persist in MSCs derived from various tissues [10,11,12,13], impacting on therapeutic efficacy and reproducibility across different studies. Consequently, greater standardisation of MSC isolation, cell culture, and characterisation of the secretome is required to improve consistency and translational reliability.

## 3. Sources of MSCs

A crucial element in clinical translation and therapeutic success is the selection of MSC sources. MSCs from various origins exhibit different features, which influence their differentiation capacity, proliferation rates, immunomodulatory profiles, and overall therapeutic efficacy. The three most common types of MSCs are bone marrow (BM), adipose tissue (AD) and umbilical cord (UC)/perinatal tissue (Table 1). Dental pulp, peripheral blood, and embryonic tissues have also been recognised as possible sources of MSCs.

Although BM was the earliest source of MSCs (BM-MSCs) [1], their acquisition is invasive, and their availability and differentiation capacity diminish with advancing age [14]. Human BM aspirates typically contain on average 0.0001% of MSCs at birth, compared to the total BM cells population, and this percentage declines with age [15]. Despite these challenges, BM-MSCs are actively utilised in orthopaedic regenerative medicine due to their differentiation capacity, as reviewed by [16]. BM-MSCs also exhibit an increased expression of vascular endothelial growth factor (VEGF), indicating a strong angiogenic potential for tissue regeneration [16] and are a reliable source for neurological regenerative therapies [17,18].

AD-derived MSCs (AD-MSCs) are widely studied in regenerative medicine applications due to their abundance and ease of collection via minimally invasive liposuction. AD-MSCs have better proliferative capacity compared to BM-MSCs, making them a good choice for generating cells for therapeutic applications, as reviewed by [19]. AD-MSCs have shown promising potential in the treatment of diabetes mellitus via endogenous pancreatic β-cell regeneration and improving insulin sensitivity, as reviewed by [20,21].

Due to their high proliferative capacity, low donor-associated variability, and robust immunomodulatory profiles, UC-derived MSCs (UC-MSCs) are widely regarded as a favourable MSC source for allogeneic applications across several diseases and disease models such as reproductive disorders, cardiovascular and neuronal conditions [22].

However, the relative suitability of UC-, BM- or AD-derived MSC-EVs is context-dependent meaning that different effects have been observed, e.g., on the proliferation of the same cell type (as reviewed by [23]).

**Table 1:** Characteristics of MSCs from different sources.

	Umbilical Cord (UC-MSCs)	Adipose Tissue (AD-MSCs)	Bone Marrow (BM-MSCs)
Tissue Source	Wharton’s Jelly, cord blood, Amniotic fluid [24]	Subcutaneous fat [25]	Iliac crest, femur marrow [6]
Collection	Non-invasive, postpartum [24]	Minimally invasive (liposuction) [25]	Invasive procedure [6]
Ethical Concerns	None [24]	Low [25]	Moderate (donor pain, consent) [6]
Yield and Proliferation Rate	High yield, fast proliferation [22]	High yield, moderate proliferation [25,26]	Moderate yield, slower proliferation [6,14]
Immunomodulation	Cytokine profile [27,28,29]	Similar to UC-MSC [29]	Similar to UC-MSC [29]
Immunogenicity	Low MHC-I, immune-privileged [30,31]	Moderate MHC-I [32]	Moderate MHC I expression [30]
Senescence and Donor Age Effect	Late senescence [24]	Increases with donor age [25,26]	Significant decline in proliferation and differentiation with age [6,14]
Storage and Banking Potential	Very commonly banked [33]	Less commonly banked	Rarely banked; mostly used fresh from extraction

Despite the initial enthusiasm, several factors still limit the use of MSCs for clinical applications, including low cell numbers and donor tissue heterogeneity. Cell senescence affecting in vitro cell expansion is another issue associated with adult MSCs. As a result, a growing number of methods now aim to obtain MSC from the differentiation of induced pluripotent stem cells (iPSCs), which represent an unlimited but homogeneous and well characterised source to produce MSCs (iPSC-derived MSCs or iMSCs). The use of iPSCs to generate MSC would also overcome ethical concerns associated with the use of human embryonic stem cells [34,35].

## 4. Extracellular Vesicles

Extracellular vesicles (EVs) are lipid-bilayer-bound particles that do not contain a nucleus and cannot replicate. Released by most cell types, they can originate from the endocytic compartment (so called exosomes), or bud directly from the plasma membrane (microvesicles or ectosomes) [36,37]. However, they are mainly categorised according to their size, into small EVs (sEVs, <200 nm) and large EVs (lEVs, >200 nm), as it is often difficult to attribute their intracellular origin accurately. EVs serve as cell-to-cell mediators by transferring bioactive molecules such as proteins, lipids and nucleic acids [38], and are involved in diverse biological processes, such as cell homeostasis, immune response, inflammation, and disease progression in pathological conditions like cancer and neurodegeneration, as reviewed by [39,40].

EV cargo is a complex mixture of different types of bioactive molecules, metabolites, and signalling molecules, often reflecting the molecular and physiological state of the parent cell/tissue [41]. Protein cargo consists of a diverse proteome, including enzymes, cell adhesion molecules and immunomodulatory factors. The microRNA (miRNA) cargo delivered by EVs have been shown to modulate gene expression through post-transcriptional modifications in recipient cells, thus exerting control over processes such as inflammation, proliferation and differentiation [42,43]. The lipid cargo not only maintains the structural integrity of the vesicles but also plays a role in cellular interactions, thus influencing the uptake of EVs by cells [44,45]. Furthermore, bioactive lipids carried by EVs can mediate vesicles functional effects [46].

According to MISEV (2018/2023) [36,47], EV studies should report key pre-analytical variables. For EV isolation from conditioned medium these include the use of EV-depleted or serum-free media to minimise contamination. EV isolation/separation methods (e.g., differential ultracentrifugation, density gradients, size-exclusion chromatography or filtration) must be fully described. Moreover, minimal characterisation requires complementary approaches: (i) particle size and concentration measurement (e.g., NTA), (ii) detection of EV-associated positive markers and appropriate contaminant negative markers (e.g., Western blotting), and (iii) imaging or single-vesicle evidence (e.g., TEM) to confirm vesicular structure.

**Figure 1:**
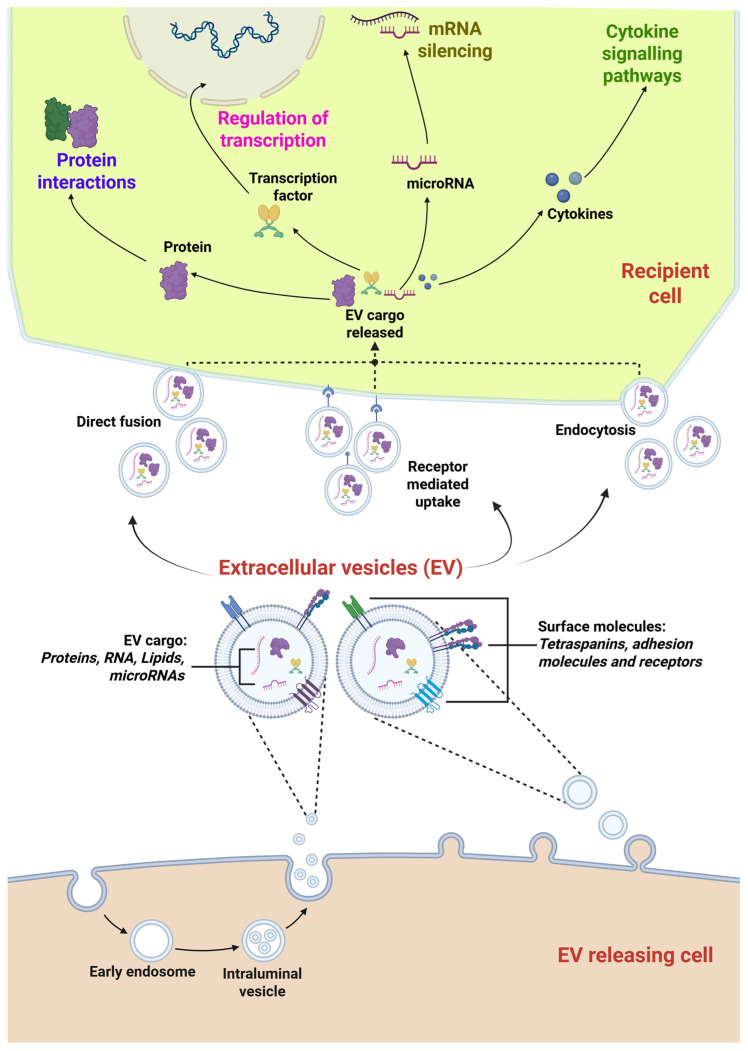
Biogenesis and paracrine effect of EVs. EVs can originate from multiple pathways. A main mechanism involves the endosomal pathway where intraluminal vesicles formed within multivesicular bodies are released into the extracellular space. EVs can also be generated by outward budding of the plasma membrane. EVs carry cell-specific biomolecules and can mediate paracrine signalling in target (recipient) cells. EVs released by MSCs can be uptaken by recipient cells through direct fusion, receptor-mediated processes or endocytosis and their cargo influence recipient-cell functions. (Figure created in Biorender, by M.J. https://BioRender.com/gsfmb92 (accessed on 30 April 2026), licenced by CC BY 4.0 [52]).

A well-described mechanism of EV biogenesis involves the endosomal system, in which vesicles form via inward budding of endosomal membranes generating intraluminal vesicles (ILV) within early endosomes, that then mature into multi-vesicular bodies (MVB). The Endosomal Sorting Complex Required for Transport machinery (ESCRT), comprising ESCRT-0, -I, -II, and -III, along with accessory proteins such as ALIX, is involved in the sorting of specific cargo associated with ILV formation [48]. In addition, ESCRT-independent pathways involving the syndecan-alix-syntenin-1 pathway also influence selective enrichment of specific proteins within these vesicles [48]. The loading of molecules and lipid structure formation is a highly regulated process, which can be affected by internal and external stimuli [48]. EVs can also arise through direct outward budding of the plasma membrane, as schematically illustrated in Figure 1.

The uptake of EVs by recipient cells is a multifaceted process that can occur through several mechanisms, being influenced by both EV-surface proteins and recipient-cell membrane receptors. Reported uptake mechanisms include direct membrane fusion, endocytosis and receptor–ligand interactions. In particular, the endocytic pathway may be clathrin-dependent and clathrin-independent, including caveolin-mediated endocytosis, macro-pinocytosis, and lipid raft-dependent internalisation [38,49]. Moreover, EVs have been reported to cross cellular barriers via transcytosis, where EVs are internalised at one membrane domain and released at the opposite site. Evidence suggests that under specific experimental conditions (e.g., alteration in barrier permeability or inflammation) EVs may cross the blood–brain barrier (BBB) [38,50]. Once EVs are internalised, the EV cargo derived from the effector cell can be released into recipient cells via mechanisms that may involve EV membrane fusion with limited membranes of endosomes or lysosomes [51], thereby influencing recipient-cell function (Figure 1).

## 5. MSCs-Derived Extracellular Vesicles and Their Cargo

EVs play an important role in mediating the paracrine effect of an effector cell from which EVs migrate to a recipient cell. The functional properties of MSC-EVs are directly linked to their unique cargo composition. Once MSC-EVs are internalised, they influence a variety of biological processes, including angiogenesis, immunomodulation, and tissue regeneration. Therefore, the analysis of MSC-EV-specific cargo is crucial for appreciating their mechanism of action and potential applications in regenerative therapy. A number of studies aimed at identifying MSC-EV specific cargo have much relied on omics approaches and RNA sequencing. This body of work has started to reveal the complexity of MSC-EV cargo enriched in specific proteome, transcriptome and lipidome. In this part of the review we aimed at searching, selecting and analysing the MSC-EV cargo published in the past 5 years. In the Supplementary file, Table S1 summarises the approach employed. One limitation of the approach was the use of PubMed as a sole search engine for MEDLINE and life-science journals; hence, some studies could potentially be missed, despite the portal covering for the vast majority of life-science journal articles.

### 5.1. Proteins as MSC-EV Cargo

A vast array of proteins from growth factors, enzymes, and cytokines to structural proteins are found enriched within MSCs according to source and culture conditions. In order to map commonly recurring proteins in published proteomic data of MSC-EV cargo, three search queries were employed to select UC, AD and BM MSC-EV proteomic data published in the last 5 years available in PubMed (Table S1 in the Supplementary file details the search methodology and Table S2 the search string). All three searches resulted in a total number of 91 articles, which were screened based on inclusion of EV proteome either in the main text or supplemental information, from research carried out in human BM-, AD- and UC-MSC-EVs. Outputs on engineered EVs or artificially cargo-loaded EVs or “secretome” with no mention of EVs were also not included. Raw proteomic data uploaded in online repositories (e.g., ProteomeXchange) were not considered, as only accessible through specialised software (Table S1 and method in the Supplementary file). Although adherence to MISEV was declared in ~30% of the selected studies (Table 2), full compliance was not consistently demonstrated. While all studies isolated EVs from serum-free conditioned media and ~65% used MISEV-recognised isolation methods with complementary EV characterisation (particle size, positive markers, and morphology), assessment of non-EV-associated markers was largely absent.

**Figure 2:**
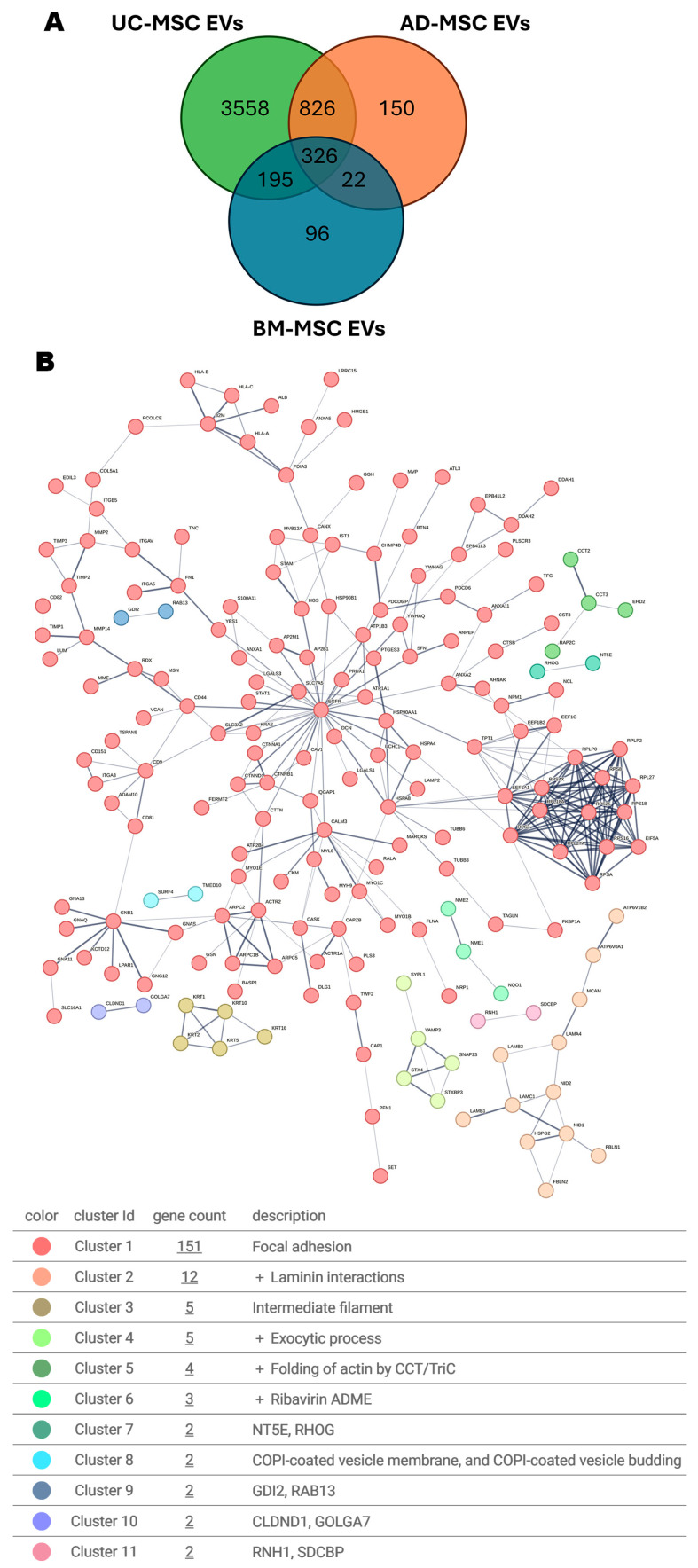
Proteomes overlap from MSC-EV cargo of different origin. (**A**) Among the MSC-EV proteomics datasets (Table 2) reported in this study (supplementary spreadsheet S1), 326 proteins were common in three MSC main sources (BM, AD and UC). (**B**). The common proteome formed a prevalent cluster (151 genes) involved in focal adhesion by STRING Lab experiments only (k mean clustering).

Most of the selected studies (Table 2) were based on comparative proteomics, evaluating differential presence of EV cargo, with only a few qualitative studies. As a consequence, the reported MSC-EV proteins were those found to be different (e.g., either between treatments or MSC types or EV isolation methods), potentially leaving out cargo not changed between the compared elements. Despite this limitation, 5173 unique proteins were extracted from the 17 proteomic datasets across 14 selected studies (Supplementary spreadsheet S1). STRING analysis based on experimentally validated interactions of AD- (1325 proteins) and BM- (640 proteins) and UC-MSC-EV proteins (randomly selected 2000 of 4906) (Supplementary spreadsheet S1), followed by k-means clustering, revealed a predominant extracellular exosome-associated cluster, thereby validating the dataset as representative EV cargo. However, substantial heterogeneity was observed in the EVs proteomes across all three MSC sources, with only about 6% common EV proteins regardless of the MSC source and methodology. Applying the same analytical strategy to the proteome shared among the three sources (326 proteins) identified a major cluster enriched for genes involved in focal adhesion (Figure 2B). Through the activation of key pathways—most notably those involving focal adhesion kinase (FAK), PI3K–AKT, and MAPK—focal adhesions promote resistance to anoikis and support cell viability in mechanically and biochemically challenging microenvironments.

**Figure 3:**
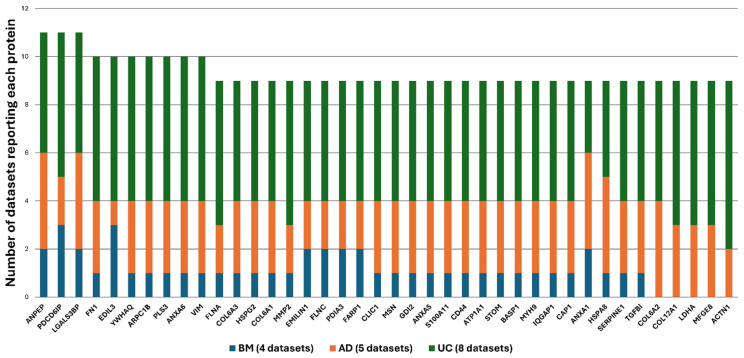
MSC-EV frequent proteome. Selected MSC-EV cargo proteins reported in at least nine proteomic datasets across MSC-EV cargo proteomic studies cited in this review (Table 2 and Table 3) and listed in Supplementary spreadsheet S1. Proteins are plotted according to their frequency of occurrence as EV cargo derived from three distinct MSC secretome sources. Bar heights reflect recurrence across studies.

Among the frequent EV proteins, about 10 were recurrent in 10+ datasets (Table 3), and 40 recurrent in more than nine studies (Figure 3) (a list of all recurrent proteins is shown in Supplementary spreadsheet S1). Among them, recurrence of several ECM proteins (fibronectin, collagens, proteoglycans and matrix metalloproteinase) was a key feature; these were consistent their crucial involvement in focal adhesion dynamics as integrin ligands. The potential in cell survival and in particular neuroprotection of this EV proteome will be evaluated in the final section of the review (Section 9). Although each study (Table 2) tested different hypotheses, common proteins in EVs originating from MSC isolated from different tissue sources were identified as shown in Figure 3. This data suggests that MSC source shapes EV composition, however a common cargo emerges in all MSC-EVs analysed regardless of the origin.

### 5.2. miRNAs as MSC-EV Cargo

miRNAs are small non-coding 20–22 nucleotide-long single-stranded RNAs that can mediate post-transcriptional gene silencing by binding to target mRNAs. Therefore, miRNAs found within MSC-EVs play a pivotal role in regulating gene expression in recipient cells contributing to their therapeutic efficacy.

**Figure 4:**
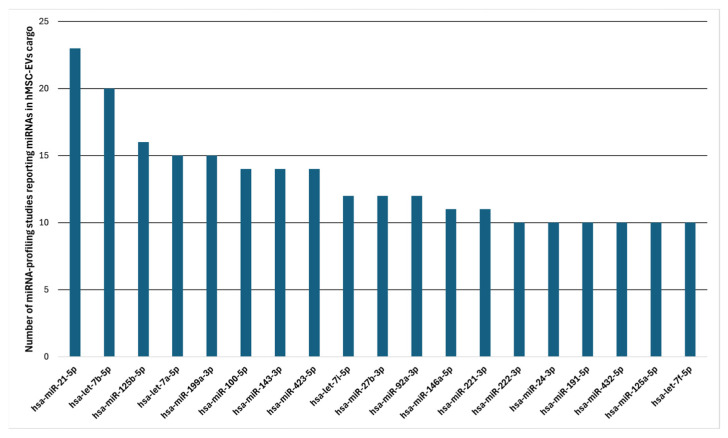
miRNAs reported in MSC-EVs. Selected MSC-EV cargo miRNAs reported in at least 10 datasets across MSC-EV cargo miRNA profiling studies cited in this review (Table 5 and Supplementary spreadsheet S2).

We have searched for the most reported miRNAs within MSC-EV cargo in PubMed in the past 5 years (Table S1 in the Supplementary file details the search methodology and Table S2 the search string). The inclusion and exclusion criteria for the search was similar to the search for proteomic datasets (Table S1 and method in the Supplementary file). The search resulted in 196 articles. Screening and selection led to 43 studies reporting differentially expressed miRNAs in human MSC-EV cargo isolated from AD, BM and UC in the most unique sources, such as menstrual blood, osteoarthritis-subchondral bone and tonsils (Table 4).

hsa-miR-21-5p emerged as the most frequently reported miRNA in at least 22 studies (Table 5 and Figure 4) (all recurrent miRNAs are listed in Supplementary spreadsheet S2). This is a highly studied miRNA with many validated and predicted gene targets: the strongest experimentally supported targets include MSLN, ADH7, RDH12 and PRKCE, involved in pro-inflammatory and immunosuppressive pathways [42,67]. hsa-miR-21-5p is downregulated in ischemic stroke and amyotrophic lateral sclerosis (ALS) [68,69] and is known to target the IL-6R gene [68]. Other miRNAs frequently present in the MSC-EV profiles were let-7 family miRNAs, known to reduce cancer stem cell renewal by acting as tumour suppressors [70]. A known target of hsa-let-7b-5p, VEGF, plays a crucial role in angiogenesis [71].

**Table 4:** MSC-EV miRNA profiling data ^∫^.

Human MSC Source	MISEV Compliance and Standards	miRNA Profile Method	miRNA Number	Approach/Scope	Citation
UC	C; NTA, TEM, WB	small RNA seq	37	Top read counts from sequencing count	[72]
UC	Declared; ultra C; NTA, FC, WB, TEM	RNA seq	16	Enriched miRNAs common between study data and 2 databases (miRNet and HDMM)	[66]
UC	Declared; ultra C; NTA, FC, WB, TEM	miRNA microarray	20	Differentially expressed miRNAs in MSC-EVs vs. ESC-EVs and iPSC-EVs	[65]
UC	Serial C; NTA, nano FC, WB	small RNA seq	13	Differentially expressed miRNAs in 2D vs. 3D MSC-EVs	[73]
BM	Serial C; NTA, TEM, WB	small RNA seq	5	Highly expressed miRNAs in MSC-EVs treated with Fe_3_O_4_	[74]
UC	Differential C; NTA, TEM, WB	small RNA seq	10	Top abundant miRNAs by read count	[75]
UC	Ultra C; nano FC, TEM, WB	RNA seq	7	No specific rank by read count or differential count	[76]
BM	Differential ultra C; NTA, TEM, WB	small RNA seq	20	Differentially expressed miRNAs in 2D vs. 3D MSC-EVs.	[77]
UC	Gradient ultra C; NTA, TEM and WB	RNA seq	11	miRNAs differentially expressed in IL-beta induced MSC-EVs	[78]
BM	Differential ultra C; NTA, TEM, WB	RNA seq	20	Top expressing miRNAs in MSC-EVs common between miRNAs from MSC as whole and MSC mimetic vesicles	[79]
AD	Tangential flow filtration; NTA, TEM	small RNA sequencing	52	Top miRNAs with most read counts	[80]
AD	Ultra C; NTA, TEM, WB	miRNA sequencing	42	Differentially expressed miRNAs in MSC-EVs from 2D and 3D cultures	[81]
IFP	Declared; step wise ultra C and CD63 immunomagnetic precipitation; NTA and FC	miRNA sequencing	24	Highly present miRNAs in relative expression to *SNORD48*	[82]
Not declared	Ultra C; TEM, NTA and Exo-check array	miRNA seq	19	19 miRNAs differentially expressed in primed vs. non-primed MSC-EVs	[83]
BM	Declared; differential ultra C; TEM, NTA, WB	miRNA expression	10	Top 10 miRNAs by read count	[84]
Chorion	Exoquick-TC; NTA, FC	small RNA seq	37	Differentially expressed miRNAs between late and early passage MSC-EVs	[85]
UC	Declared; Exo-spin, NTA, WB	RNA seq	60	Differentially expressed miRNAs in senescent vs. non-senescent MSC-EVs	[86]
UC	Declared; sequential ultra C; NTA, TEM, WB	small RNA seq	10	Most enriched miRNAs by unique molecular indices	[87]
Olfactory mucosa	Declared; differential ultra C; TEM, NTA, WB	miRNA microarray	19	Differentially expressed miRNAs in hypoxic vs. normoxic MSC-EVs	[88]
IFP	Anion exchange chromatography and ultrafiltration; TEM, NTA, WB	small RNA seq	25	Top miRNA by total read count	[89]
MSC544 cell line	Declared; differential ultra C; NTA, WB	TaqMan Advanced miRNA Human AB-Card	25	Top miRNA by appearance	[90]
IFP	Declared; step-wise ultra C; NTA, TEM, FC	Predesigned hMSC exosome 166 miRNA qPCR	48 and 82	Top miRNAs of two priming conditions of MSC-EVs	[91]
AD and UC	C and ultrafiltration; NTA, TEM and WB	small RNA sequencing	41	Differentially expressed miRNAs in two MSC-EV sources vs. stem cell-EVs	[92]
SHED, UC, AD, BM	C and filtration; NTA	Human 3D-gene microarray	50	Top expressed miRNAs in four sources of MSC-EVs	[93]
Amniotic fluid	Concentration and ultra C; NTA, ELISA	TaqMan low density arrays pool A	23	Differentially expressed miRNAs in 2nd vs. 3rd trimester amniotic fluid MSC-EVs	[94]
Labial gland	Sequential C with Amicon centrifugal filters; NTA, TEM, WB	miRNA sequencing	10	Differentially expressed miRNAs in Sjögren’s syndrome vs. healthy control MSC-EVs	[95]
Wharton jelly (UC)	Declared; ultra C and Exoquick-TC; NTA, TEM, WB	TaqMan Low-Density Array	24	Top highly expressed miRNAs in MSC-EVs based on Ct-values	[71]
Palatine tonsil	Ultra C; Exo-Quick; NTA, TEM, and WB	RNA sequencing	10	Top miRNAs by read count in MSC-EVs	[96]
UC	PEG precipitation, ultra C, Exoquick; NTA, TEM, WB	miRNA sequencing	15	Sequenced miRNAs reported for their biological function	[97]
Cartilage and subchondral bone	Declared; ultra C; NTA, TEM, FC	miRNA expression assay	48	Differentially expressed miRNAs in osteoarthritis vs. healthy MSC-EVs	[98]
Placenta	Declared; ultra C; NTA, TEM, WB	miRNA microarray	40	Differentially expressed miRNAs in irradiated vs. non irradiated MSC-EVs	[99]
AD	Declared; ultra C; NTA, TEM, WB	miRNA seq	9	Differentially expressed miRNA in obese vs. lean AD-MSC-EVs	[100]
BM	Ultra C; NTA, TEM, nano FC	RNA seq	25	Top miRNAs by read count in MSC-EVs	[67]
BM	Declared; ultra C; NTA, TEM, WB	small RNA seq	15	Top miRNAs in MSC-EVs by read count	[101]
AD	Declared; ultra C and PEG treatment; NTA, TEM, nano FC	small RNA sequencing	25	Top miRNAs in MSC-EVs by transcripts per million	[102]
UC	C and ExoEasy kit; TEM, NTA, WB	small RNA sequencing	8	Top miRNA in MSC-EVs by relative miRNA expression level	[89]
Cornea and BM	Declared; ultra C, precipitation; NTA, Exoview, TEM, WB	small RNA seq	26	Differentially expressed miRNA in cornea- vs. BM MSC-EVs and 2D vs. 3D	[103]
Placenta	C and Exosome RNA purification kit; TEM, WB	RNA seq	30	Top expressing miRNAs in MSC-EVs by fold change	[104]
UC	Declared; tangential flow filtration and ultra C; TEM, Nano FC	miRNA seq	40	Top abundant miRNAs by fold change	[105]
UC	QIAGEN exosome isolation kit; NTA	miRNA seq	28	Top miRNAs in MSC-EVs by counts per million	[106]
Menstrual blood	Ultra C; TEM, NTA, WB	miRNA seq	70	Differentially expressed miRNAs in primed vs. non primed MSC-EVs	[107]
UC	EV trap exosome extraction kit; NTA, TEM, WB	miRNA seq	12	Top expressed miRNAs by transcripts by million	[108]
iPSC derived	Ultra C; NTA, FC	miRNA seq	9	Top abundant miRNAs normalised by read counts	[109]

^∫^ Data extracted from PubMed deposited search articles from 15 February 2021–15 February 2026. UC—umbilical cord; AD—adipose; BM—bone marrow; IFP—infrapatellar fat pad, SHED—stem cells from human exfoliated deciduous teeth; iPSC—induced pluripotent stem cells; C—centrifugation; TEM—transmission electron microscopy; NTA—nanoparticle tracking analysis; WB—Western blotting; FC—flow cytometry.

**Table 5:** miRNA reported in at least 10 independent miRNA datasets across MSC-EV cargo literature ^∫^.

miRNA	Citation
hsa-miR-21-5p	[65,67,71,72,73,76,79,82,84,89,90], [91] *, [94,95,97,101,102,103,104,105,106,109]
hsa-let-7b-5p	[65,67,71,74,75,76,79,84,87,89,91,95,96,98,101,102,103,104,105,106]
hsa-miR-125b-5p	[71,72,73,75,84,86,87,89,90,91,94,95,101,105,106,109,110]
hsa-let-7a-5p	[67,72,76,79,84,89,91,95,99,101,102,103,105,106,111]
hsa-miR-199a-3p	[65,67,71,72,80,84,86,87,89,90,91,94,95,101,106]
hsa-miR-100-5p	[65,67,71,72,73,75,79,89,91,92,97,101,103,104]
hsa-miR-143-3p	[67,72,74,89,90,92,94,95,101,103,104,105,106,109]
hsa-miR-423-5p	[65,67,72,75,85,89,90,91,94,98,104,105,107]
hsa-let-7i-5p	[65,67,72,79,84,89,95,101,103,104,105,106]
hsa-miR-27b-3p	[65,67,79,82,91,98,101,103,104,106,109]
hsa-miR-92a-3p	[65,67,71,72,79,89,94,102,103,104,105,109]
hsa-miR-146a-5p	[65,80,81,86,92,97,99,102,105,106,107]
hsa-miR-221-3p	[65,67,71,72,90,91,94,103,105,106,109]
hsa-miR-222-3p	[67,71,72,77,81,85,89,94,98,104]
hsa-miR-24-3p	[67,71,86,89,94,96,98,102,106,107,110]
hsa-miR-191-5p	[65,71,72,75,79,96,98,101,105,106]
hsa-miR-432-5p	[67,73,80,86,87], [91] *, [104,105,107]
hsa-miR-125a-5p	[73,75,79,80,90,94,97,98,105,106]
hsa-let-7f-5p	[67,72,79,91,95,98,101,102,103,105,106]

^∫^ Data extracted from PubMed deposited research articles from 15 February 2021–15 February 2026. * This citation miRNA lists the miRNA in two different experimental quantification.

### 5.3. Lipids as MSC-EV Cargo

Lipids are the structural backbone of EVs, driving their formation, cargo sorting, survival in the ECM and biofluids, and fusion with recipient cells. Despite their pivotal role, only a limited number of lipidomic studies have investigated the composition of MSC-EV lipid cargo. Based on PubMed, a database search for MSC-EV lipidomic data (Table S1 in the Supplementary file and method; Table S2 for search string) revealed a total of 14 studies out of which, one was a duplicate and seven studies were either non-MSC, non-lipid or only targeting selective lipids, and, therefore, were discarded from further scrutiny. The six remaining studies had a full lipidomic profiling of MSC-EV cargo and are listed in Table 6.

In a first comprehensive study, Haraszti et al. [44] compared EV cargo from BM-MSCs with that of cancer lines (U87 glioblastoma and Huh7 hepatocellular carcinoma), identifying 1961 lipid species across 22 lipid classes. BM-MSC-EVs were significantly enriched in glycolipids, free fatty acids, phosphatidylserines (PS), and, notably, cardiolipins (CL), which were commonly enriched in both MSCs and Huh7 hepatocellular carcinoma cells [44]. In a separate study, Haraszti et al. also demonstrated that the lipid profiles dynamically changed by cellular stress and priming conditions [112]. CL, phospholipids specifically present in the inner mitochondrial membrane and required for optimal OXPHOS, were the only significantly enriched class of lipids, with the dilysocardiolipin subclass showing the highest enrichment [112]. Combined hypoxia and cytokine priming resulted in elevated lipid intensities and in particular membrane phospholipids phosphatidylcholine (PC) and phosphatidylethanolamine (PE) displayed the highest increase in MSC-EVs [113]. Bismonoacylglycerophosphate (BMP), a lipid biomarker for endosomally derived vesicles, was highly enriched in small EVs [113].

Using dental pulp MSC (DP-MSCs)-EVs, isolated from three independent donors, Amaro-Prellezo et al. identified PC, sphingomyelins and triglycerides as dominant lipids in DP-MSC-EVs [114]. Lipidomic profiling of Wharton’s Jelly (WJ) MSCs (WJ-MSCs) also revealed that phospholipids were the dominant class of lipids, with PC being the most abundant among all phospholipids [115]. Cholesterol esters were the second highest lipid component, followed by ceramides [115]. While PC, PE and sphingomyelins were dominant in two different preparations of MSC-EVs isolated from the decidual placenta (maternal interface) and the chorionic placenta (foetal interface) cells [116]. The presence of CL in the decidual placenta related well with the high oxidative stress environment of the maternal side of the placenta [116]. Table 6 summarises this MSC-EV lipidome analysis.

## 6. EVs Derived from MSCs as a Cell-Free Approach in Regenerative Medicine

Because of their cell signalling properties, ability to cross biological barriers and bioengineering potential, EVs have been increasingly researched for their therapeutic potential, especially in the scope of regenerative medicine as carriers for delivering bioactive molecules [117]. In PubMed the number of publications on advances in MSC-EVs has increased by almost 10 times in the past 10 years.

Among the advantages of MSC-derived EVs compared to whole cell MSCs are that EVs are non-self-replicating, have minimal immunogenicity and often do not express the major histocompatibility complex, therefore they offer a safer approach than straight cell therapy, reducing the risks that come with cell engraftment [118,119,120]. Unlike direct infusion of MSCs via intravenous delivery, MSC-EVs are less likely to cause embolisms due their nano-scale size, which facilitate the crossing of biological barriers such as the BBB [119]. Moreover, EVs display enhanced biodistribution at sites of injuries; for example in the kidney, accumulating specifically at wound sites [121] and ameliorating kidney disease through multiple mechanisms, as reviewed by [117,122].

Pre-clinical studies using animal models have demonstrated the therapeutic potential of MSC-EVs in various disease conditions. In cardiac regeneration, using a mouse model of myocardial infarction, Koo et al. demonstrated that MSC-EV treatment improved functional outcomes and enhanced cardiac tissue remodelling [123]. This therapeutic effect was largely attributed to the payload of miRNAs, proteins, and lipids encapsulated within MSC-EVs, which modulate inflammatory responses, prevent cardiomyocyte apoptosis, and stimulate resident cardiac progenitor cells. Las Heras et al. demonstrated the potential of MSC-EVs in chronic wound healing by promoting proliferation and survival of skin cells [120]. In a mild metabolic osteoarthritis model, MSC-EVs displayed regenerative properties and promoted cartilage regeneration, suggesting a potentially more beneficial therapeutic strategy than direct MSC-based approaches [124]. In models of spinal cord injury, administration of BM-MSC-EVs promoted recovery by improving the integrity of the blood-spinal cord barrier (BSCB) through the expression of tight junction proteins like ZO-1, occludin and claudin-5 [125]. In foetal brain injury models, MSC-EVs protected the brain after hypoxia-ischemia [119].

Halting neurodegeneration is an urgent priority in a field with still limited mechanistic understanding. In recent years, the potential of MSCs and MSC-EVs to counteract neurodegeneration has been explored with growing interest, although the number of published studies remains limited compared with other therapeutic fields. In this review we aim to further explore this emerging area. The subsequent sections will examine the current state of research on MSC-derived EVs in neuroprotection and their emerging therapeutic potential.

## 7. MSC-EVs in Neuroprotection

Neuroprotection refers to treatments aimed at preserving the structure, function, and viability of neurons to reduce the rate of neuronal loss during neurodegeneration [126] the effects of which are seen in Alzheimer’s disease (ADis), Parkinson’s disease (PDis), Huntington’s disease, and ALS. Although distinct in terms of different aetiologies, progression and pathological hallmarks—such as beta-amyloid plaques and tau protein in ADis, and α-synuclein aggregates in PDis, these conditions are characterised by a progressive loss of neuronal function driven by shared mechanisms involving neuroinflammation, protein aggregation, metabolic stress, dysfunctional neurovascular coupling, and by the influence of both environmental and genetic factors [127].

Neuroprotective strategies encompass interventions that mitigate convergent mechanisms of injury, common across neurodegenerative diseases [127]. They include boosting neurons’ intrinsic resilience mechanisms (i.e., molecular chaperons, the ubiquitin-proteasome system and autophagy), enhancing mitochondrial function, antioxidative and anti-inflammatory interventions, the modulation of excitotoxicity, neurotrophic support and the promotion of neuronal repair and regeneration.

In preclinical studies, MSC-EVs have been reported to promote neuroprotection, neuro-restoration and functional rescue through different mechanisms and proved effectiveness in different pathological contexts including ADis, PDis, ALS, Multiple Sclerosis (MS, a neuroinflammatory disease with a neurodegenerative component) and acute neurodegenerative events, like the ones associated with brain injury and ischemia. Starting from these lines of evidence, we will analyse how MSC-EVs can influence the critical molecular and cellular events underlying neurodegeneration.

Neuroinflammation is a key player in the onset and progression of neurodegenerative diseases. By inheriting the renowned immunomodulatory properties of their donor cells, MSC-derived EVs can confer neuroprotective effects by localising to sites of inflammation [128,129] and interacting with cells involved in inflammatory processes—such as microglia, astrocytes, and peripheral immune cells like T cells—promoting an anti-inflammatory environment [130]. They can inhibit the release of pro-inflammatory cytokines, such as IL-6 and TNF-α, and promote the production of anti-inflammatory cytokines, including IL-10. Indeed, adipose MSCs have been reported to preferentially accumulate at inflammatory lesions associated with experimental autoimmune encephalomyelitis (EAE, experimental model of MS), reducing T cell infiltration, cytokine release, demyelination and axonal damage [128]. In line with these observations, intranasal administration of MSC-EV induced immunomodulatory responses and ameliorated EAE clinical score [131]. In addition, EVs from hypoxia-preconditioned BM-MSCs have been reported to attenuate spinal cord inflammation and injury in rats by polarising macrophages towards a pro-regenerative phenotype via vesicular miR-146a-5p [132]. Accordingly, in an APP/PS1 ADis mouse model, EVs from hypoxia-preconditioned MSCs suppressed inflammation, thus counteracting cognitive decline via an increase in brain miR-21 [133]. Similarly, MSC-EVs engineered to express miRNAs that target NF-κB signalling have had positive effects in traumatic brain injury by suppressing neuroinflammation [134]. Furthermore, MSC-EVs have been proved effective in carrying therapeutic agents (such as Donepezil) for brain-targeted drug delivery against neuroinflammation in experiments on zebrafish larvae [135].

Mitochondrial dysfunction, elevated oxidative stress, and disrupted metabolic homeostasis are central processes driving neurodegenerative pathology. Adipose MSCs can restore mitochondrial function in ALS in vitro models [136,137]. In ADis, mitochondria can be transferred from human UC-MSCs to cells as EV cargo [138], similarly to what MSCs do through nanotubes (tube-like bridges connecting cells) and other mechanisms [139] to restore ATP and mitigate beta-amyloid and tau pathology. EVs are abundant in antioxidant enzymes and compounds, including catalases, superoxide dismutase (SOD) and glutathione peroxidase [140]. Importantly, catalase delivered as MSC-EV-cargo have been shown to protect hippocampal neurons from oxidative stress and synaptic damage induced by beta-amyloid oligomers [141,142]. MSC-EV antioxidant effects have been reported in various in vitro models of oxidative stress [143], including the 6-hydroxy-dopamine (6-OHDA) dopaminergic neurons PD model [144] and motorneuron-like ALS models [145]. This was proven in vivo in alcohol-induced brain damage [146], and brain ischemic injury, where MSC-mediated neuroprotection has been linked with miR-132-3p vesicular content [147].

Neurons are energetically demanding and more sensitive to stress than most cell types and apoptosis is one of the major mechanisms of neuronal death when survival pathways fail. MSC-EVs can also suppress apoptosis via various routes. They can control intracellular apoptotic signalling pathways, like PI3K-Akt- and Bcl-2-related pathways [148], and contain heat shock proteins (HSP), which can bind and inhibit apoptotic proteins. MSC-EVs carry miRNAs involved in the anti-apoptotic mechanism, such as miR-21 which inhibits the expression of apoptotic proteins [149]. In a PD context, MSC-EV cargo has been proven to protect dopaminergic neurons from cell death [150]. This may be mediated by induced autophagy to inhibit apoptosis [151]. Similarly, a specific type of EVs termed micro-electrical-fields-induced MSC-EVs—released by MSCs after controlled electrical stimulation as a new therapeutic approach for neuro-regeneration and repair—counteracted apoptosis by activating autophagy via lncRNA MALAT1/miR-22-3p/SIRT1/AMPK axis in spinal cord injury [152]. Additionally, MSC-EVs have shown neurorestorative functions, e.g., after spinal cord injury [152] and stroke [115]. This is considered with the fact that MSC-EVs carry neurotrophic molecules like brain-derived neurotrophic factor (BDNF) and nerve growth factor (NGF) [153,154] that stimulate neuronal differentiation.

In the brain, the ECM is heavily enriched in glycoproteins and proteoglycans and it is characteristically more hydrated and softer, conferring stability and protection to neurons. ECM components such as laminin and fibronectin support neural synapse formation by providing structural scaffolding, guiding axonal growth, and contributing to the repair of peripheral nerve injuries. Through the formation of specialised perineuronal nets (PNNs), the ECM helps protect neurons against oxidative stress and controls plasticity. Therefore, when the ECM is disrupted, neurons become more vulnerable to cell death. In recent reports, MSC-EVs have been shown to promote ECM remodelling by stimulating the synthesis of ECM components, particularly collagen, elastin, and fibronectin, and by regulating metalloproteinases [155]. For instance, MSC-EVs enriched in miR-27b regulate ECM remodelling via the ITCH/JUNB/IRE1α pathway, accelerating cutaneous wound healing [156]. In addition, MSCs secretome (including EVs) has been reported to drive ECM remodelling by acting on microglia [157]. In agreement with these observations, tail-vein injection of MSC-EVs supported the restoration of hippocampal neuronal morphology and function in APP/PS1 ADis mice [158]. Similarly, MSC-EVs have been shown to stimulate neurogenesis and favour cognitive recovery in a mouse model of ADis induced by Aβ1–42 aggregates injection into the dentate gyrus [159]. Interestingly, by delivering VEGF, fibroblast growth factor (FGF), and other angiogenic molecules, MSC-EVs could boost neuro-restoration by facilitating angiogenesis, providing essential nutrients and oxygen to neurons in affected areas [160].

In a number of studies, MSC-EVs exhibited targeted functions against disease-specific protein aggregates through different mechanisms. For example, MSC-EVs displayed the ability to phagocytose or bind protein aggregates. Furthermore, MSC-EVs have been shown to carry high levels of Aβ-degrading enzymes, such as neprilysin (NEP) and insulin-degrading enzyme (IDE) [161]. EVs from human UC-MSCs significantly upregulated these Aβ-degrading enzymes, thereby reducing Aβ deposition in APP/PS1 transgenic mice and improving cognitive function [33]. Similarly, adipose MSC-EVs controlled the aggregation of the pathological SOD1 protein restoring the levels of mitochondrial proteins in neurons from G93A-mutated ALS mice [162].

One of the major challenges for the development of therapies against neurodegenerative diseases is the presence of the BBB, a highly specialised structure composed of endothelial cells which form the brain microvasculature, surrounded by pericytes and astrocytes endfeet. The barrier is tightly closed thanks to the presence of several complexes that form between the endothelial cells monolayer, such as tight junctions and adherence junctions [163]. The potential of EV-based therapies to naturally cross the BBB, make EVs an ideal drug delivery system targeting the CNS.

## 8. Delivery Routes to Brain and MSC-EV Based Clinical Trials

Growing experimental evidence suggests that some EV subtypes can cross barriers via receptor-mediated transcytosis, i.e., internalisation by endocytosis, followed by MVB formation and consequent exocytosis. This has been specifically demonstrated in a few in vitro and in vivo studies showing that some EVs populations (e.g., plasma-derived EVs and tumour-EVs) can be internalised by endothelial cells crossing the BBB under pro-inflammatory conditions [164,165,166] or as part of the metastatic process [167]. As described in a previous section of this review, several pre-clinical studies have demonstrated the efficacy of MSC-EVs in neuroprotection, especially in comparison to stem cell therapies, hence paving the way for initial human clinical trials.

Currently, seven clinical trials using MSC-EVs are registered on ClinicalTrials.gov or the International Clinical Trials Registry Platform (ICTRP) (accessed on 1 February 2026) for treatment of neurodegenerative diseases, of which only one is completed, three are still in the recruiting phase, two are suspended and one has unknown status (Table 7). Initial results from these trials suggested that MSC-EV-based biologics are well tolerated, safe, can be effectively administrated intranasally or via IV-injections, and show some promising clinical neuroprotective effects in multiple neurodegenerative diseases, although further studies are required to confirm clear clinical benefits and to clarify the underlying biological mechanisms driving their therapeutic action. In ADis, trial NCT04388982 is testing intranasal administration of allogenic adipose-MSC-EVs (ahaMSCExos) in three dose groups, reporting no adverse events, improved cognitive functions in the medium-dose arm (4 × 10^8^ particles, equal to 10 µg MSCs-Exos) maintained up to week 36, trend decrease in hippocampal atrophy, with nasal delivery well tolerated [168]. This study focused mainly in assessing the preliminary safety and efficacy of the MSC-EVs-based treatment in ADis cohorts and was therefore based on a small sample size (i.e., N = 3 per group) with no control group. Other limitations concern the selected inclusion criteria, which did not account for confounding effects such as different age at disease onset, comorbidities and variable ADis stages. The authors also suggest that future trial designs should include either dose escalation or titrations to evaluate the maximum tolerated dose for the ahaMSCs-Exos, which was not contemplated in the current study. Although early indications of cognitive improvements were shown, the biological effects post-treatment were modest and not statistically significant, possibly due to the low sample size. Trial NCT07105371 (completed in 2025) used intranasal UC-MSC-derived EVs (AlloEx) on a broad motor-disorders focused cohort (ALS, KD, CMS, LBD) and found no adverse events and modest clinical improvements in nearly all patients, with some benefits lasting up to six months [169]. In this study the sample size was higher, but the included cohorts were not equally distributed among disease groups, with an overrepresentation of ALS patients compared to the other motor disorders. The absence of statistical analysis and prevalence of qualitative self-reported data also limits the significance of these findings. Nevertheless, the clinical ameliorations observed in some patients (higher ALSFRS-R scores and reduced muscle stiffness in the ALS cohort; trend toward decreased motor impairment in the LBD cohort) remain noteworthy, as the authors report this as the first evidence of symptom improvement and functional strengthening in these diseases following therapy. The same team is responsible for trial NCT05152394, which is currently evaluating the safety of intranasal AlloEx for treatment of PD. Although no official outcomes have been reported yet, a preprint manuscript shows that ~80% of participants displayed some decrement in symptoms up to one year after treatment with consequent increase in quality of life [170]. As for the previous trial, statistical analysis is not included, and many of the data shown refer to only a sub-group of the patients, not the whole cohort. The results currently presented in the pre-print document are very encouraging, but in the absence of peer-review the effectiveness of the AlloEx treatment cannot yet be robustly assessed, and additional studies are required to determine whether it constitutes a truly disease-modifying therapy for PD. Similarly, recent trials are also focusing on UC-MSC-EVs nasal drop therapy to treat either ALS (NCT06598202, recruitment ongoing) or different neurodegenerative disorders more broadly (NCT06607900, not yet recruiting), with data due to be shared from 2026 to 2028. The remaining trials have been suspended due to slow enrolment (NCT07146087) or pandemic-related issues (NCT04202770), with no results published.

Although additional research and time are needed to draw definite conclusions, emerging evidence from these clinical trials strongly indicates that MSC-EVs hold great promise as novel therapeutics, with the potential to provide cross-disease neuroprotection in line with their established anti-inflammatory, anti-apoptotic, anti-oxidative and neurorestorative properties.

## 9. Decoding the Power of MSC-EV Cargo in Neuroprotection

Despite encouraging findings from preclinical studies and early clinical trials (Table 7), MSC-EV based interventions targeting neurodegeneration still encounter significant challenges related to MSC heterogeneity and poor control of the secretome, raising concerns on long term safety and potential adverse effects. In this review we mapped MSC-EV specific cargo, found in the literature, in terms or proteins, miRNA and lipids. Despite variability in aim and research questions among different studies, and limitations in data retrieval outlined in the approach (Table S1), a number of molecules have emerged as recurrent cargo in work deposited in PubMed in the past 5 years (Table 3, Table 5 and Table 6). Here their possible role in neuroprotection is discussed.

Among the top 10 cited MSC-EV proteins, aminopeptidase N (ANPEP) was reported in several studies as cargo in BM-, AD- and UC-MSC-EVs. Indeed, the soluble form of ANPEP increased brain microglial activation and proinflammatory cytokine expression in a mouse model of neuroinflammation, and mechanistically, astrocytic ANPEP was shown to interact with the microglial proinflammatory receptor angiotensin type 1 receptor [171]. However, like brain-specific aminopeptidases (e.g., puromycin sensitive aminopeptidase, PSA [172]), ANPEP, which also cleaves neutral amino acids at the N terminal end of peptides, could potentially contribute to degrade toxic aggregates such as tau tangles, as initially reported for PSA [173]. Although the involvement of PSA could not be confirmed [174] this hypothesis is intriguing, and it is tempting to speculate that MSC-EV-associated ANPEP could help degrade toxic aggregates, as previously suggested. Programmed cell death 6-interacting protein (PDCD6IP), known as apoptosis-linked gene-2-interacting protein 1 (ALIX) [175], was another recurrent cargo across MSC-EVs from different sources; it is also a “marker” of EVs being a core ESCRT-associated protein driving intraluminal vesicles formation inside MVBs, and other functions tied to the EV formation. Indeed, its presence is normally correlated with neuroinflammation and neuronal death [176]. Consistent with this, PDCD6IP was found downregulated in MSCs from differentiated cells in a direct comparative proteomic study [65]. Another recurrent cargo in MSC-EV literature was galectin-3 binding protein (LGALS3BP) which on the contrary was found upregulated in the UC-MSC-EVs versus iPSC/embryonic EVs in the same study [65]. A secreted protein, interacting with tetraspanin EV markers and many members of the ECM and ECM receptors, LGALS3BP is enriched in human neuronal progenitor cells and it is secreted by EVs, playing an important role in human corticogenesis by modifying the extracellular environment; individuals with *LGALS3BP de novo* variants exhibited altered folding of the cerebral cortex [177]. Although there is no direct evidence supporting its involvement in the regenerative potential of MSC-EVs, LGALS3BP could plausibly contribute to neuronal maintenance. Among other highly recurrent MSC-EV proteins, fibronectin (FN1) was repeatedly shown to support neuroprotection, primarily by promoting neuronal survival [178]. Heparan sulfate (HS) proteglycan perlecan (HSPG2), a large proteoglycan with negatively charged HS chains, and a major structural component of cerebral basement membranes, regulating pericytes and stabilising the BBB [179], was also recurrent in MSC-EVs. The HS chains of HPG2 are known to associate with well characterised neurotrophic factors (e.g., FGF2 and other pro-survival factors such as TGM2) [180]. As a frequent surface cargo of MSC-EVs, HSPG2 is regarded as a “docking” platform for other HS binding proteins (such as FN, TGM2) and can contribute to neuronal survival and repair. If confirmed in targeted studies, the presence of four collagen subtypes among recurrent MSC-EV cargo (COL6A3, COL6A1, COL6A2, COL12A1), together with FN, HSPG2, and matrix metalloproteinase (MMP2), underscores the potential importance of ECM proteins in mediating the regenerative properties of MSC secretome. This is consistent with our data reporting the specific effect of MSC secretome on microglia-mediated ECM remodelling [157]. The ECM glycoprotein EGF like repeats and discoidin domains 3 (EDIL3), a highly adhesive molecule frequently found in brain and lung [181], is also highly cited in MSC-EV literature. Despite being suggested to negatively affect dendritic spine dynamics in APOE4 astrocytes in co-culture with primary neurons, and to accumulate in postmortem APOE4 ADis brains [182], EDIL3 is also known to have anti-leukocyte recruitment activity/anti-inflammatory properties; in a number of inflammation-related pathologies, it promoted endothelial cells adhesion through the alpha-v/beta-3 integrin receptor, inhibiting formation of vascular-like structures [183]. Thus, EDIL3 represents another MSC-EV candidate for further investigation. Several other MSC-EV proteins highly cited in the literature are implicated in neuronal survival, including annexin A6 (ANXA6), a Ca^2+^ dependent membrane repair protein that helps seal damaged neuronal membranes. ANXA6 has been involved in synaptic plasticity and neuronal resilience after injury [132] and protects against ADis in a mouse model [184]. YWHAQ, belonging to the 14-3-3 protein family and frequently found in MSC-EVs, is known for binding phosphoserine-containing proteins and for regulating survival pathways. It is expressed in many brain regions, including the substantia nigra. Multiple studies have shown that 14-3-3 proteins protect neurons by stabilising pro survival signalling pathways, preventing apoptosis through binding BAD, and supporting dopaminergic neuron development [185]. Among other recurrent MSC-EV cargo, filamin A (FLNA), plays a key role in neuronal migration and synaptic connectivity [186]. Vimentin (VIM) is a type III intermediate filament cytoskeleton protein recurrent in MSC-EVs, as it can also be fully externalised (e.g., via a non-classical EV-mediated secretion pathway) [187]. The effects of VIM once externalised can be contrasting but are beneficial in injury recovery. For example, astrocyte-secreted VIM promoted *in vitro* axonal growth and recovery in mice with spinal cord injury [188,189]. Plastin 3 (PLS3), an F-actin-bundling protein plays major roles in actin cytoskeleton organisation and axonal growth and stability, with a well-defined neuroprotective function in motor neurons. PLS3 has been reported to restore key processes depending on actin dynamics in spinal muscular atrophy (SMA) neurons [190]. Actin-related protein complex subunit 1B (ARPC1B), part of the ARP2/3 complex, controlling actin polymerization in the human body, has been involved as a centrosomal protein in mitosis, and it emerged as highly cited in MSC-EV proteomics. Fundamentally an immune-system actin-regulation protein, ARPC1B is mainly expressed in hematopoietic cells [191], and many tumour regulating genes are highly connected with it. Recent work has also highlighted a possible anti-inflammatory effect for ARPC1B, as strong predisposition to inflammatory diseases is present in ARPC1B human deficiency [192]. On the contrary, moesin (MSN) has not been shown to have neuroprotective effects, and evidence seem to link moesin to neurodegeneration, especially in ADis, where it contributes to tau-induced neurotoxicity, actin overstabilization, and microglial activation [193].

Among miRNA cargo (Table 5), let-7b-5p was frequently reported in MSC-EVs. Let-*7* family members are among the most abundantly expressed miRNAs in brain [194] and the miRNA let-7b is a ligand for toll-like receptor (TLR) 7 in microglia and neurons. TLR 7 is involved in various forms of CNS injury, including classical neurodegenerative diseases. However, let-7b-5p also targets genes involved in proliferation (*Ras* MAPK) stemness and inflammation. It was found downregulated in EVs from MS patients [195]. Therefore, let-7b-5p rich MSC-EVs could potentially reduce inflammation. Interestingly, mouse let-7b-5p was found to be overexpressed in a recent study analysing the cargo carried by EVs released from immortalised mouse hippocampal neuronal cells (HN9.10) following ceramide stimulation, a known inducer of EV secretion; mouse let-7b-5p-regulated gene targets include those involved with sphingolipids metabolism/function and neuronal development [196], suggesting a “positive” role in neurons. As let-7b-5p was largely found in UC-MSC-EV compared to iPSC-EVs and embryonic stem cells EVs [65], it is tempting to speculate that its presence may be linked with a “mature mesenchymal” signature. Another recurrent miRNA in MSC-EV literature was hsa-miR-21-5p, the 5′-arm mature miRNA produced from the human *MIR21* gene. Regarded as a survival miRNA, it is typically upregulated in response to oxidative stress, hypoxia and inflammation, inhibiting the expression of pro-apoptotic genes [197]. Treatment with small EVs isolated from WJ-MSCs, including-miR-21-5p and let-7 family (as well as miR-22-3p and miR-27b-3p), triggered oligodendrocyte maturation and reduced oxygen-glucose deprivation-mediated neuronal apoptosis typical of premature white matter injury [198].

In summary, surveying recent literature on MSC-EV cargo has revealed a consistent set of molecules that appear to underlie the anti-inflammatory and tissue-repair properties commonly attributed to MSC-EVs across multiple sources.

## 10. Conclusions

The last few years have marked a transition from purely symptomatic management of neurodegenerative diseases to a new era of disease-modifying therapies, such as those driven by advancement in monoclonal antibodies. The introduction of Lecanemab and Donanemab monoclonal antibodies targeting beta-amyloid plaques has signed a turning point in ADis therapy [199,200]. Still, these treatments are approved only for patients at early disease stages and slow down the disease without being able to stop it, with longer term outcomes currently unknown and no improvement of cognitive functions expected. Therefore, a cure for neurodegenerative disease is still lacking, and the search for novel therapeutic approaches remains essential.

Several studies have shown evidence of neuroprotection and neuro-regeneration by MSCs of different origin in neurodegenerative disorders [201]. EVs released by MSCs represent a very promising cell-free intervention that has proven to be immunomodulatory, antioxidant, anti-apoptotic and regenerative [154] and may overcome some of the limitations connected to cell-therapies.

Current challenges relate to several factors including poor standardisation of the procedures, unpredictability of the cultures and variations between the secretomes. The possibility to generate iMSC from iPSC is opening the way to more reproducible and stable cultures [202].

As most of the therapeutic potential of MSCs is mediated through their EVs, this review set out to capture the essence of EV cargo by surveying recent literature. Several limitations emerged: not all published datasets were fully accessible, and we did not stratify secretome profiles according to treatment conditions. Consequently, our analysis encompasses MSC-EV secretomes derived from adult stem cells of diverse origins, examined both at baseline and under a variety of stimuli, without distinction.

However, a compelling pattern emerged. MSC-EVs consistently carried anti-inflammatory and immunomodulatory proteins, miRNAs with immune-regulatory potential, and extracellular matrix components associated with cell survival. Individually or synergistically, these cargo elements may contribute to processes associated with neuro-regeneration.

Mapping the MSC-EV cargo has helped clarify this complex and rapidly evolving field. The neuroprotective potential highlighted across recent studies offers promising avenues for therapeutic development in an area where progress remains limited, despite an urgent societal need.

## Figures and Tables

**Table 2:** MSC-EV proteomic data ^∫^.

Human MSC Source	MISEV Compliance and Standards	Proteomics Method (Analysis)	EV Input	Proteome Size (Identified Protein Number)	Approach/Scope	Citation
BM and UC	Declared; 1-step sucrose C; NTA, TEM, WB	LC-MS/MS; LFQ (Progenesis, “Ion Accounting” algorithm/UniProt)	1 µg	264 (49) ^ϒ^–BM 732 (119)–UC	Differentially expressed proteins between hypoxic and normoxic MSCs	[53]
BM	Ultra C; NTA, TEM, WB	LC-MS/MS; LFQ (Proteome discoverer, MASCOT/UniProtKB)	500 ng	701 (175)	Comparative proteomics between osteogenic EVs and naïve-EVs	[54]
BM	Ultra C; NTA, WB	As above	500 ng	LC-MS/MS913 (444)	Differential expressed proteins between hypoxic and normoxic MSCs	[55]
BM	Density gradient-Ultra C; polymer-based precipitation (Exoquick), SEC; NTA, TEM, DLS	LC-MS/MS (Proteome discoverer/UniProt)	3 µg	102 (31)	Comparative proteomics of differently isolated EVs (ultra-C and SEC)	[56]
AD	SEC; NTA, WB	Tandem mass- tag and LC-MS/MS (Proteome discoverer/UniProt)		139 (41)	Differentially expressed proteins between hypoxic and normoxic MSCs	[57]
AD	Declared; polymer-based precipitation (Exoquick^TM^), NTA, TEM, WB	iTRAQ and LC-MS/MS (Proteome discoverer, MASCOT/Swiss-Prot)	25 µg	1190 (168)	Differential expression between hypoxic lipopolysaccharide-stimulated and non-stimulated MSCs	[58]
AD and UC	C and filtration; NTA, WB	iTRAQ and LC-MS/MS (Proteome discoverer, MASCOT)		1695 (315)–AD (362)–UC	Differential expression between two sources (UC and adipose tissue)	[59]
AD	Ultra C, polymer-based precipitation (Exoquick); SEC; NTA, DLS, TEM	iTRAQ and LC-MS/MS (Proteome discoverer, MASCOT/Swiss-Prot)	25 µg	14,691 (599)	Comparative proteomics of AD-MSC-EVs obtained from different EV isolation methods	[60]
UC	Declared; serial filtration; NTA, WB	LC-MS/MS (analyses by Biotools, Taiwan)	200 µg	(14)	Relative protein abundance score	[61]
UC	Ultra C; TEM, WB	LC-MS/MS diaPASEF data and DIA-NN analysis in library-free mode (UniProtKB/Swiss-Prot)	10 µg	4200 (4200)	Quantitative proteomic analysis of UC- vs. BM-MSC-EVs	[62]
UC	Ultra C; NTA, TEM, WB	Not disclosed MS; IBAQ (intensity-basedabsolute quantification)		(1350)	Absolute quantification	[63]
UC and AD	Ultra C; NTA, TEM, WB	LC-MS		(724)–AD (1246)–UC	Qualitative evaluation of AD- and UC-MSC-EVs	[64]
UC	Declared; ultra C; NTA, TEM, WB	LC-MS/MS; tandem mass tag labelling and LFQ (Proteome discoverer, MASCOT)		(276)	Differential expression in EVS from iPSCs, embryonic stem cells and MSCs	[65]
UC	Declared; ultra C, filtrations; FC, NTA, TEM, WB	LC-MS/MS; LFQ (Maxquant software V1.6.17.0/UniProt)	4 × 10^9^ EV particles	(420)	Qualitative analysis of UC-MSC-EV cargo from different donors	[66]

^∫^ Data extracted from PubMed deposited research articles from 12 January 2021–12 January 2026. ^ϒ^ In parenthesis the number of proteins identified by mass spectrometry. UC—umbilical cord; AD—adipose; BM—bone marrow; C—centrifugation; NTA—nanoparticle tracking analysis; DLS—dynamic light scattering; TEM—transmission electron microscopy; WB—Western blotting; FC—flow cytometry; iTRAQ—Isobaric tags for relative and absolute quantitation; LFQ—label free quantification.

**Table 3:** Proteins reported in at least 10 independent proteomic datasets across MSC-EV cargo literature ^∫^.

Protein	Citation
ANPEP	[55,56,58,59,60,62,63], [64] *, [65,66]
PDCD6IP/ALIX	[54,55,56,59,60,62,63], [64] *, [65,66]
LGALS3BP	[55,56,58,59,60,62,63], [64] *, [65,66]
FN1	[54,58,60,61,62,63], [64] *, [65,66]
EDIL3	[54,55,56,59,62,63], [64] *, [65,66]
YWHAQ	[55], [59] *, [60,62,63], [64] *, [65,66]
ARPC1B	[55,57,59,60,62,63], [64] *, [65,66]
PLS3	[55,57,59,60,62,63], [64] *, [65,66]
ANXA6	[53,55,59,60,62,63], [64] *, [65,66]
VIM	[53,55,59,60,62,63], [64] *, [65,66]

^∫^ Data extracted from PubMed deposited research articles from 12 January 2021–12 January 2026. * This citation has two datasets one of AD-MSC-EVs and one of UC-MSC-EVs.

**Table 6:** Most reported lipids in lipidomic datasets across literature on MSC-EV cargo ^∫^.

Human MSC Source	MISEV Compliance and Standards	Lipidomic Method	Lipid	Approach	Citation
BM	Differential C; TEM, NTA, WB	LC-MS/MS	Phosphatidylserines, glycolipids, free fatty acids, cardiolipins, and lysophosphatidyl serines	Comparative proteomics of BM-MSC-EVs vs. U87 and Huh7 cancer cell lines	[44]
UC	Differential C; TEM, NTA, WB	LC-MS/MS	Cardiolipins	Proteomics of UC-MSC-EVs upon cellular stress and priming	[112]
AD	2-step ultra C; NTA; FC; TEM	LC-MS	Bismonoacylglycerophosphate (BMP), phosphatidylethanolamine, phosphatidylglycerol, phosphatidylethanolamine, diacylglycerol, phosphatidylcholine, alkyl ether-linked phosphatidylcholine, and sphingomyelin	Proteomics of AD-MSC-EVs upon cellular stress and priming	[113]
Dental pulp	Declared; tangential flow filtration and SEC; TEM, NTA, WB	LC-MS	Phosphatidylcholine, sphingomyelins, and triglycerides	Proteomics of DP-MSC-EVs from three independent donors	[114]
Wharton Jelly (UC)	Declared; tangential flow filtration; TEM, cryo-EM, ELISA, nano FC, WB	LC-MS	Phosphatidylcholine, phosphatidylserines, sphingomyelins, cholesterol esters, and ceramides	Proteomics of WJ-MSC-EVs	[115]
Placenta—Decidua and chorion	Declared; LEAP chromatography; cryo-TEM, NTA, WB	LC-MS	Cardiolipin, phosphatidylcholine, phosphatidylethanolamine, sphingomyelins, and phosphatidylserines	Proteomics of two different MSC populations derived from placenta	[116]

^∫^ Data extracted from PubMed deposited research articles from 15 February 2021–15 February 2026. UC—umbilical cord; AD—adipose; BM—bone marrow; C—centrifugation; NTA—nanoparticle tracking analysis; TEM—transmission electron microscopy, WB—Western blotting, FC—flow cytometry.

**Table 7:** Clinical trials involving the use of MSC-EVs as biologicals for the treatment of neurodegenerative ^∫^.

Disease	Trial ID/Link *	Title and Sponsor/Location	EV Source	Route/Dose	Phase	Status	Key Outcomes/Related Publications
ADis	NCT04388982	The Safety and the Efficacy Evaluation of Allogenic Adipose MSC-Exos in Patients with Alzheimer’s Disease Ruijin Hospital Shanghai, China	Allogenic adipose-derived MSC exosomes * (ahaMSC-Exos)	Intranasal administration; twice a week for 12 weeks; three dosage groups: 5, 10 and 20 µg per administration N = 9 (3 per group)	I/II	Uknown	Safety met, no adverse effects reported; medium-dose arm showed ADAS-cognitive improvement at week 12 and maintained to week 36; nasal drip well tolerated [168]
ALS, KD, CMS and LBD (motor disorders)	NCT07105371	Patients With ALS and Other Motor Disorders Will be Treated with Mesenchymal Cell Exosome Solution The Foundation for Orthopaedics and Regenerative Medicine Antigua and Barbuda	UC-MSC exosomes (AlloEx)	Intranasal administration; single-arm non-controlled study; five patient groups, receiving from 1 up to 2.5 doses of AlloEx (on one or two consecutive days) N = 18	I	Completed (2025)	No adverse events reported; all patients achieved some degree of clinical and strength improvement (apart for one); longest improvement recorded at the 6-month follow-up [169]
PDis	NCT05152394	Safety of Cultured Allogeneic Adult Umbilical Cord Derived Mesenchymal Stem Cell Exosomes for Parkinson’s Disease The Foundation for Orthopaedics and Regenerative Medicine Antigua and Barbuda	UC-MSC exosomes (AlloEx)	Intranasal administration; two back-to-back daily doses of 4 cc each (~8 × 10^11^ exosomes); single group assignment N = 19	I	Recruiting	Unknown [170]
ALS	NCT06598202	Exploring Nasal Drop Therapy with Small Extracellular Vesicles for ALS (de-ALS) Xuanwu Hospital, Beijing, China	UC-MSC-derived exosomes (hUC-MSC-sEV-001)	Intranasal administration; Once daily, twice a week, for a total of two weeks; multicentre, randomised, double-blind, placebo-controlled, dose-escalation trial	I-II	Recruiting	Unknown
Neurodegenerative diseases, (ADis, PDis, MSA, LBD, FTD, ALS)	NCT06607900	hUC-MSC-sEV-001 Nasal Drops for Neurodegenerative Diseases Xuanwu Hospital, Beijing, China	UC-MSC-derived small EVs (hUC-MSC-sEV-001)	Intranasal administration; multi-centre, open, single-arm, basket-design clinical trial	I	Not yet recruiting	NA
PMS	NCT07146087	Allogeneic Mesenchymal Stem Cell-Derived Exosome Therapy for Progressive Multiple Sclerosis Biocells Medical Warsaw, Poland	Allogenic BM-MSC-derived exosomes	Intravenous infusions; every 3 months for 1 year (total 4 doses) treatment vs. placebo group.	Early Phase I	Suspended (slow enrolment)	NA
Depressio, anxiety, and neurodegenerative dementia	NCT04202770	Focused Ultrasound and Exosomes to Treat Depression, Anxiety, and Dementias Neurological Associates of West Los Angeles Santa Monica, CA (US)	Allogenic amniotic fluid derived exosomes	Intravenous infusion of unconcentrated exosomes solution (15cc, equivalent to 21 million stem cells) after 1 h of transcranial focused ultrasound targeting the hippocampus	NA	Suspended (pending COVID-19 pandemic; pending status of product development)	Unknown

ADis: Alzheimer’s disease; PDis: Parkinson’s disease; ALS: amyotrophic lateral sclerosis; KD: Kennedy disease; CMS: Congenital myasthenic syndrome; LBD: Lewy body dementia; MSA: multiple system atrophy; FTD: frontotemporal dementia; PMS: progressive multiple sclerosis. ^∫^ Data extracted from ClinicalTrials.gov up to 1 February 2026. * The term “exosome” was retained in this Table if present in the clinical trial titles or study details.

## Data Availability

No new data were created or analysed in this study.

## Supplementary Materials

The following supporting information can be downloaded at: https://www.mdpi.com/article/10.3390/cells15100889/s1, Supplementary file_Search method and limitations.docx; Spreadsheet S1_MSC-EV cargo proteins.xlsx; Spreadsheet S2_MSC-EV cargo miRNA.xlsx.

## Abbreviations

The following abbreviations are used in this manuscript:

MSCs	Mesenchymal Stromal Cells
AD	Adipose tissue
UC	Umbilical cord
BM	Bone Marrow
ST	Stromal Tissue
WJ	Wharton’s Jelly
EV	Extracellular vesicles
ECM	Extracellular matrix
ADis	Alzheimer disease
PDis	Parkinson disease
ALS	amyotrophic lateral sclerosis
CL	Cardiolipin
