# Supplementary material for: Uncovering the Secret of Mesenchymal Stromal Cells Secretome: From Extracellular Vesicle Cargo to Neuroprotection
Source: Cells. 2026 May 13;15(10):889. doi: 10.3390/cells15100889 (PMC13204574; doi:10.3390/cells15100889)
Supplement: Supplementary file 1 [file cells-15-00889-s001.zip › Supplementary File_search methods and limitations.pdf]

Supplementary File

METHOD (For section 5.1 to 5.3 of the review article)

ELIGIBILITY CRITERIA The study characteristics employed to include a study of MSC-EV proteome were as follows:

(1) Year of dissemination: 12th Jan 2021 - 12th Jan 2026 for proteomic study search, 15th Feb 2021 - 15th Feb 2026 for miRNA study search, any date until the 15th of Feb 2026 for lipidomic study search; (2) language: English; (3) report status: full research articles, published; (4) content: for MSC EVs proteome, human AD, BM, UC as MSC sources; for MSC-EVs miRNA and lipids, any human MSC source for MSC-EVs; (5) inclusion of protein/miRNA/lipid data either in the main text or supplemental information (not raw proteomic data uploaded in external on line repositories).

SOURCES Original journal articles found in databases accessible through the PubMed search engine.

SEARCH STRATEGY To search for articles with data on MSC-EV cargo (proteomes, miRNomes, lipidomes) a specific search string strategy was employed (displayed in supplementary Table S2). The search strings were created with a limitation to exclude Review articles.

SELECTION PROCESS A single reviewer carried out the screening of the included studies in consultation with a supervisory team of three people, based on the inclusion and exclusion criteria. The resultant articles from the search strategy were exported to Rayyan online software (only employed as an abstract manager for the ease of reading and screening of abstracts). Following the initial screening, the papers were exported as full text versions and were read through manually by the single reviewer as a secondary screening, to further refine the final selection of articles based on the inclusion and exclusion criteria that could only be assessed by reading through the entire article.

DATA COLLECTION PROCESS

After the secondary screening and selection of final articles, proteomic data, miRNA data and lipidomic data were extracted from the main text or supplementary files of the article. Data were initially collected by one reviewer and confirmed by a second investigator (100% of the proteomic studies; 50% miRNA and 100% lipid studies). Disagreements were resolved by discussion. Inconsistencies between reports were resolved by consulting a local proteomics specialist.

DATA ITEMS (outcomes) Data were collected for three macromolecules (proteins, miRNAs and lipids). Protein IDs emerged from protein studies were included as standard gene symbols and miRNA IDs from miRNA profiling studies were included as standard

miRNA names (according to miRBase), when listed as MSC-EV cargo in either qualitative or comparative studies, provided they appeared in the publication or in the supplementary data in form of tables, spreadsheets or in heatmaps. Lipid classes and sub-classes were similarly extracted as class names in the main text, or as tables or in heatmaps, when showed as MSC-EV cargo.

STUDY RISK OF BIAS ASSESSMENT PubMed, a search engine for MEDLINE and life-science journals was the only portal employed to retrieve MSC-EV cargo, hence potentially some studies could be missed. However, this is unlikely, as PubMed covers the vast majority of molecular biology, genetics, biochemistry, and related life-science journal articles.

A limitation is that most proteomic studies were comparative hence reporting protein IDs of MSC-EV cargo found to be different between treatments or MSC types or methodologies for EV isolation, potentially leaving out cargo not changed between the compared elements.

Table S1: Search methodology, data collection and limitations (For sections 5.1 – 5.3 of the review article)

**Eligibility**			
	Year of dissemination	Report types:	Content
Proteome	12th Jan 2021- 12th Jan 2026	Published (no pre-prints) full research articles	human AD, BM, UC as MSC sources
miRNAs	15th Feb 2021– 15th Feb 2026	As above	Any human MSC type
Lipidome	Any date until – 15th of Feb 2026	As above	Any human MSC type
**Sources**			
**Proteome/ miRNAs/Lipidome**	Databases accessible through the PubMed search engine – searching for all journal articles except reviews		
**Search Strategy**			
**Proteome/ miRNAs/Lipidome**	Each omics approach had their unique search string. Full search string in Table S2		
**Selection**			
**Proteome/ miRNAs/Lipidome**	Single reviewer screened the papers for selection based on inclusion and exclusion criteria following the search		
**Data collection**			
Proteome	Single reviewer involved in selection data and a second reviewer confirmed 100% of proteomic studies selected by first reviewer		
miRNAs	Single reviewer involved in selection data and a second reviewer confirmed 50% of miRNA studies selected by first reviewer		
Lipidome	Single reviewer involved in selection data and a second reviewer confirmed 100% of lipidomic studies selected by first reviewer		
**Outcomes**			
	Data result	Study type:	Data content
Proteome	Protein IDs extracted as Gene symbol from MSC-EV cargo proteome data provided in the study	Qualitative or comparative proteomic studies	Shown in the main or in supplementary information (no external repositories)
miRNAs	miRNA IDs extracted from MSC-EV cargo miRNAs in the form	Qualitative or comparative transcriptomic studies	Shown in the main or in supplementary information (no

	of standard miRBase naming conventions		external repositories)
Lipidomes	Lipid names extracted as lipid classes and sub-classes from MSC-EV lipidomic studies	Qualitative or comparative lipidomic studies	Shown in main or supplementary information (no external repositories)
**Limitations /bias**			
**Proteome/ miRNAs/Lipidome**	Most studies are comparative approaches – cargo although specific to MSC-EV cargo but detected in comparison to specific controls or cell culture conditions or EV isolation methods		
**Search strategy**	Single literature search engine		

Table S2 PUBMED search

**Search term for proteomic studies**			**Search term for miRNA profiling studies**			**Search terms for lipid profiling studies**	
**Human Umbilical cord MSC-EVs**	(("perinatal" OR "Umbilical cord") AND ("mesenchymal*") AND ("Extracellular vesicles" OR "Exosome") AND ("Proteomic" OR "Proteome")) NOT (review[Title/Abstract]).		**All human sources for MSC-EVs**	(((human Mesenchymal*) AND (extracellular vesicles OR exosomes)) AND (miRNA profil* OR microRNA profil*)) NOT (review[Title/Abstract]).		**All human sources for MSC-EVs**	(("mesenchymal*") AND ("Extracellular vesicles" OR "Exosome") AND ("Lipidome" OR "Lipidomic")) NOT (review[Title/Abstract]).
**Human Adipose tissue MSC-EVs**	(("Adipose") AND ("mesenchymal*") AND ("Extracellular vesicles" OR "Exosome") AND ("Proteomic" OR "Proteome")) NOT (review[Title/Abstract]).						
**Human Bone marrow MSC-EVs**	(("Bone-marrow") AND ("mesenchymal*") AND ("Extracellular vesicles" OR "Exosome") AND ("Proteomic" OR "Proteome")) NOT (review[Title/Abstract]).						

Flow diagrams for article search and selection (Sections 5.1-5.3 of the review article)

(1)

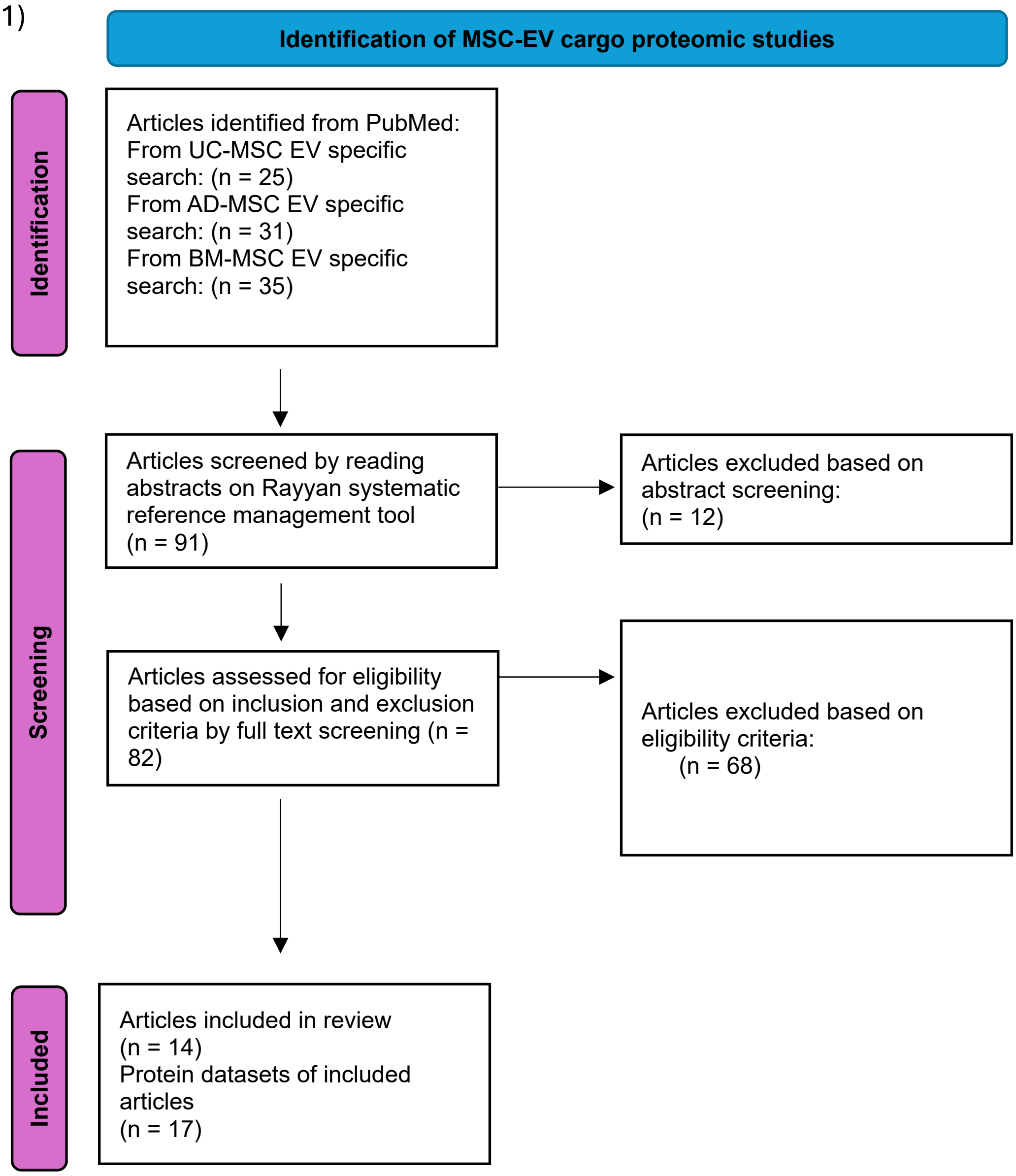

(2)

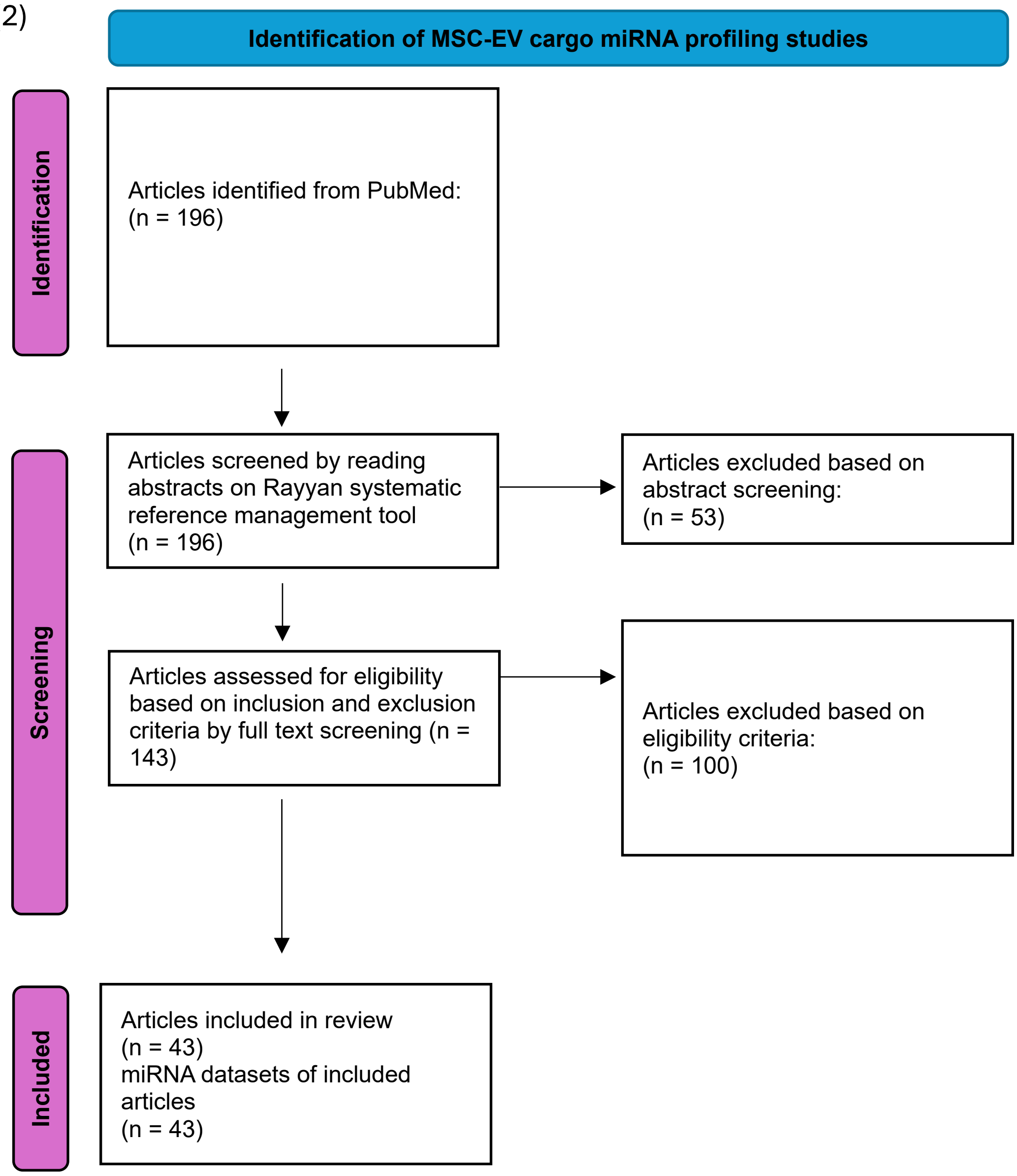
Identification of MSC-EV cargo miRNA profiling studies
Identification
Articles identified from PubMed: (n = 196)
Screening
Articles screened by reading abstracts on Rayyan systematic reference management tool (n = 196)
Articles excluded based on abstract screening: (n = 53)
Articles assessed for eligibility based on inclusion and exclusion criteria by full text screening (n = 143)
Articles excluded based on eligibility criteria: (n = 100)
Included
Articles included in review (n = 43)
miRNA datasets of included articles (n = 43)

(3)

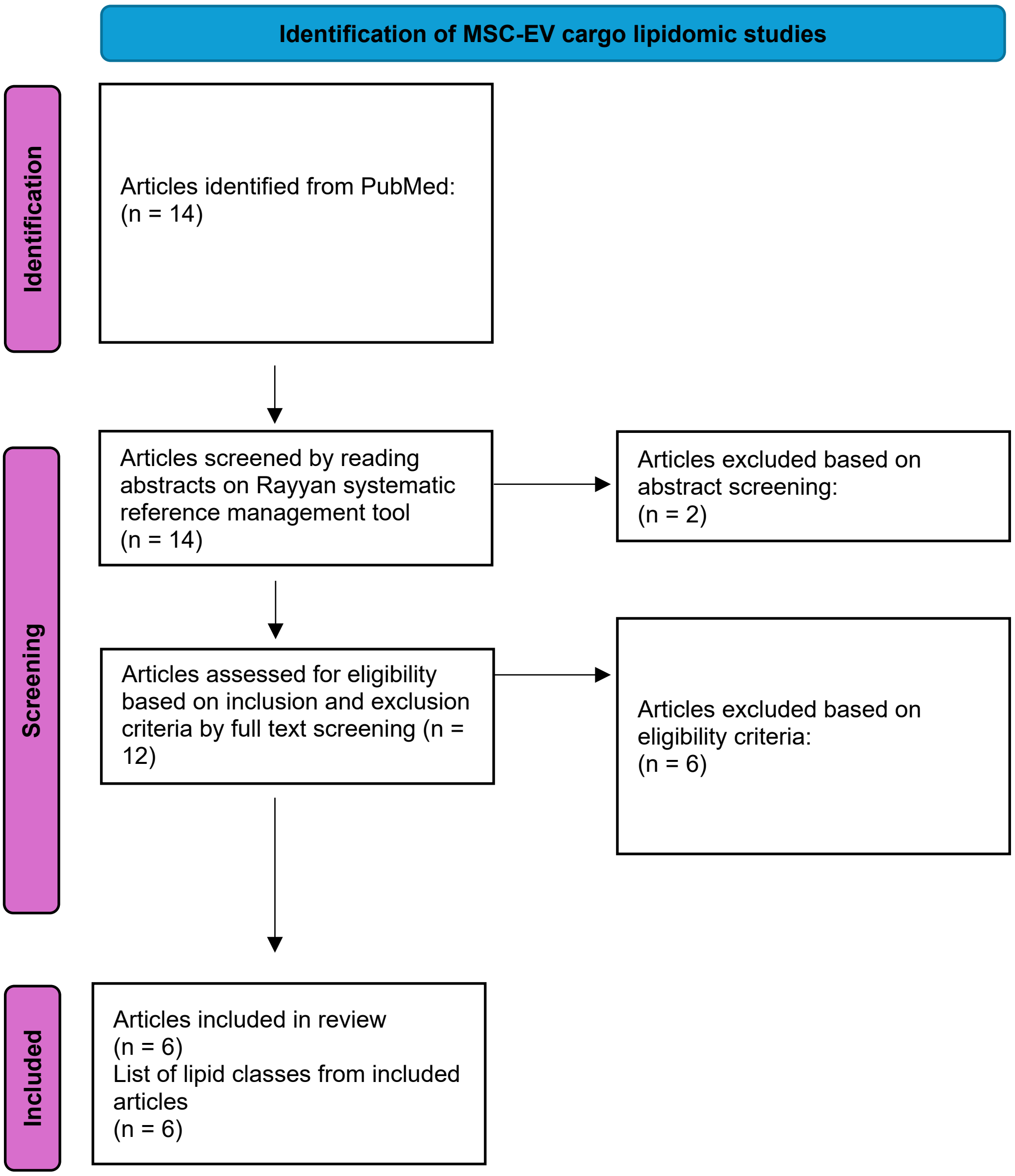
Identification of MSC-EV cargo lipidomic studies
Identification
Articles identified from PubMed: (n = 14)
Screening
Articles screened by reading abstracts on Rayyan systematic reference management tool (n = 14)
Articles excluded based on abstract screening: (n = 2)
Articles assessed for eligibility based on inclusion and exclusion criteria by full text screening (n = 12)
Articles excluded based on eligibility criteria: (n = 6)
Included
Articles included in review (n = 6) List of lipid classes from included articles (n = 6)
